# Reducing antimicrobial use in livestock alone may be not sufficient to reduce antimicrobial resistance among human *Campylobacter* infections: an ecological study in the Netherlands

**DOI:** 10.1017/S0950268824001511

**Published:** 2024-11-27

**Authors:** Huifang Deng, Linda E. Chanamé Pinedo, Anouk P. Meijs, Pim Sanders, Kees T. Veldman, Michael S. M. Brouwer, Wieke Altorf-van der Kuil, Bart Wullings, ISIS-AR Study Group, Maaike J. C. van den Beld, Sabine C. de Greeff, Cindy M. Dierikx, Engeline van Duijkeren, Eelco Franz, Lapo Mughini-Gras, Roan Pijnacker

**Affiliations:** 1 Institute for Risk Assessment Sciences, Utrecht University, Utrecht, the Netherlands; 2 Centre for Infectious Disease Control, National Institute for Public Health and the Environment (RIVM), Bilthoven, the Netherlands; 3 The Netherlands Veterinary Medicines Authority, Utrecht, the Netherlands; 4 Wageningen Bioveterinary Research, part of Wageningen University and Research, Lelystad, The Netherlands; 5 Wageningen Food Safety Research, Wageningen, the Netherlands

**Keywords:** antimicrobial resistance, antimicrobial use, *C. jejuni*, *Campylobacter coli*, one health

## Abstract

Reducing antimicrobial use (AMU) in livestock may be one of the keys to limit the emergence of antimicrobial resistance (AMR) in bacterial populations, including zoonotic pathogens. This study assessed the temporal association between AMU in livestock and AMR among *Campylobacter* isolates from human infections in the Netherlands between 2004 – 2020. Moreover, the associations between AMU and AMR in livestock and between AMR in livestock and AMR in human isolates were assessed. AMU and AMR data per antimicrobial class (tetracyclines, macrolides and fluoroquinolones) for *Campylobacter jejuni* and *Campylobacter coli* from poultry, cattle, and human patients were retrieved from national surveillance programs. Associations were assessed using logistic regression and the Spearman correlation test. Overall, there was an increasing trend in AMR among human *C. jejuni*/*coli* isolates during the study period, which contrasted with a decreasing trend in livestock AMU. In addition, stable trends in AMR in broilers were observed. No significant associations were observed between AMU and AMR in domestically produced broilers. Moderate to strong positive correlations were found between the yearly prevalence of AMR in broiler and human isolates. Reducing AMU in Dutch livestock alone may therefore not be sufficient to tackle the growing problem of AMR in *Campylobacter* among human cases in the Netherlands. More insight is needed regarding the population genetics and the evolutionary processes involved in resistance and fitness among *Campylobacter.*

- Reduced livestock AMU was not associated with reduced AMR in *Campylobacter jejuni*/*coli* isolates from human cases.
- AMU in broiler was not associated with AMR among *C. jejuni*/*coli* isolates in broiler samples at slaughter.
- Higher AMR among broiler *C. jejuni*/*coli* isolates was associated with higher AMR among *C. jejuni*/*coli* isolates from human cases.

## Introduction

Human campylobacteriosis is the most frequently reported zoonosis in Europe, with *C. jejuni* causing over 90% of reported cases and *C. coli* being responsible for most of the remaining cases. In the Netherlands, campylobacteriosis is associated with the highest disease burden among 14 food-related pathogens (3,300 DALY), with an estimated cost-of-illness of 67 million euros in 2019 [1]. Although most *Campylobacter* infections cause self-limiting gastrointestinal illness, antimicrobial therapy may be needed for severe or prolonged infections. Infections with antimicrobial-resistant *Campylobacter* spp. are harder to treat and may result in prolonged hospital stays, treatment failure, increased risk of severe illness, and healthcare costs [2]. In recent years, however, *Campylobacter* infections resistant to clinically relevant antibiotics have become increasingly prevalent, particularly infections resistant to fluoroquinolones. A monitoring study conducted in the United States of America showed that *C. coli* isolates had a higher prevalence of resistance to most of the examined antimicrobials as compared to *C. jejuni* isolates from chicken and turkey [3]. In the EU, high to extremely high levels of resistance to critically important antimicrobials for the treatment of *Campylobacter* infections in humans were reported from humans and animals. Particularly, resistance to ciprofloxacin in *C. jejuni* from humans increased in 12 reporting countries over the period 2013 – 2021, and the cause of this trend is unclear [4].

There is growing evidence suggesting that antimicrobial use (AMU) in livestock selects for antimicrobial resistance (AMR) among their bacterial populations, which can be transmitted to humans [5-7]. In the Netherlands, successful policies have been enforced to reduce AMU in livestock. These policies were applied mainly after the country was ranked as one of the highest consumers of antibiotics among European Union (EU) countries in 2007 [8]. National surveillance reports indicated that the sales of antibiotics in 2022 decreased by 77.4% compared to the reference year of 2009 [9]. While it is clear that reducing the amount of antimicrobials consumed by livestock contributes to reducing AMR among indicator bacteria in these animals [10-12], it is still unclear to what extent reducing AMU in animals has beneficial effects on reducing the prevalence of AMR among certain human infections of zoonotic origin, such as *Campylobacter.*

The main aim of this study was to assess (1) the association between AMU in the main livestock sources of human campylobacteriosis in the Netherlands (i.e., poultry and cattle) and AMR among *C. jejuni/coli* isolates from human campylobacteriosis cases in the Netherlands over the period 2004 – 2020. Additional analyses were performed to assess (2) the association between AMU and AMR among *C. jejuni/coli* isolates from livestock and (3) the association between AMR in *C. jejuni/coli* isolates from livestock and AMR among isolates from human campylobacteriosis cases. This study focused on antimicrobials of relevance for clinical treatment of campylobacteriosis, i.e., tetracyclines, macrolides, and fluoroquinolones.

## Materials and methods

### Antimicrobial use and resistance data

For data on AMU in livestock, we focused on antibiotic usage in poultry and cattle because they are the main livestock sources of campylobacteriosis in the Netherlands [13]. The annual defined daily dose per animal per year (DDDA/Y) was retrieved for broilers (2004–2020), turkeys (2013–2020), veal calves (2007–2020), dairy cattle (2004–2020), and other cattle (2012–2019). Both total AMU and AMU of specific antimicrobial classes (Anatomic Therapeutic Chemical veterinary level 3rd/4th, https://www.whocc.no/atcvet/atcvet/) were included. AMU data from 2004 – 2011 were collected by Wageningen Economic Research [14], and between 2012 – 2020 by the Netherlands Veterinary Medicines Authority (SDa, www.autoriteitdiergeneesmiddelen.nl) [15]. AMU data from the Wageningen Economic Research was based on a selected number of farms and were weighted to represent each animal sector nationwide. Details are available on the LEI website (www.lei.wur.nl). Measurements from SDa were on the national level.

Data on AMR per antimicrobial agent class (tetracyclines, macrolides and fluoroquinolones) for *C. jejuni* and *C. coli* in poultry (broilers 2004–2020, and turkeys 2011–2012) and cattle (calves 2006–2012, dairy cattle 2010–2012, and other cattle 2006–2009) in the Netherlands were obtained from Wageningen Bioveterinary Research (WBVR, Lelystad, the Netherlands). All isolates were obtained from the national AMR monitoring program, and they were collected at slaughter level according to European legislation [16]. Data on AMR in clinical human cases (2004–2020) were obtained from the laboratory surveillance system at the National Institute for Public Health and the Environment for the period 2004 – 2013, with a national coverage of 52% [17]. For the period 2014 – 2020, data from the Dutch Infectious Diseases Surveillance Information System for Antimicrobial Resistance (ISIS-AR) was used, with a national coverage of 64% [9, 18]. For the first, the outcome of AMR as interpreted by the laboratories was used, while for the latter, minimum inhibitory concentration (MIC) and inhibition zone diameters were used to interpret the samples as resistant, intermediate, or susceptible based on the European Committee on Antimicrobial Susceptibility Testing (EUCAST) clinical breakpoints version 10.0; 2020 (www.eucast.org). Isolates with an intermediate susceptibility were included in the analysis combined with the resistant category. AMR (number of resistant isolates over the total number of tested isolates per year) and AMU (DDDA/Y) data were matched on year and antimicrobial class and combined as one dataset.

### Statistical analysis

Logistic regression models for aggregated AMR data were used to assess the association between AMU in livestock and AMR in isolates from human campylobacteriosis cases (association 1), with separate models per animal sector (broilers, turkeys, and veal calves), antibiotic class (tetracyclines, macrolides and fluoroquinolones), and *Campylobacter* species (*C. jejuni* and *C. coli*). To account for potential co-selection effects, the total usage of antimicrobials other than the one under study was included in the logistic regression models. Multicollinearity among explanatory variables was checked using variance inflator factor (VIF), and variables were excluded if the VIF score was larger than five. Results were expressed as odds ratios with corresponding 95% confidence intervals (95% CI). In a sensitivity analysis, the effect of a one-year lag of antimicrobial usage on resistance was explored for the association between AMU in livestock and AMR in *C. jejuni/coli* from human cases. Similar logistic regression (including potential co-selection adjustment and multicollinearity check) was used to assess the association between AMU and AMR in *Campylobacter* isolates from livestock per antimicrobial class and *Campylobacter* species (association 2). Additionally, the correlations between annual AMR prevalence in isolates from livestock and annual AMR prevalence in isolates from humans (with a one-year lag) per antimicrobial class and per *Campylobacter* species were explored using a Spearman correlation test (association 3). For all analyses, only antimicrobial classes with DDDA/Y ≥ 0.5 in each year were assessed to obtain reliable estimates from the models. All analyses were performed in R Studio version 4.2.0 (R Foundation for Statistical Computing, Vienna, Austria).

## Results

### Descriptive results

**Figure 1.**
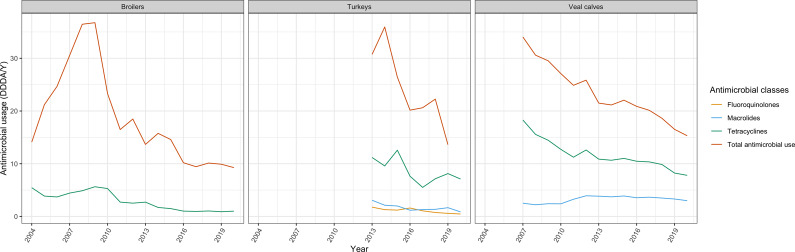
Antimicrobial usage (defined daily dosages per animal per year, DDDA/Y) in broilers, turkeys, and veal calves in the Netherlands (2004–2020). * AMU retrieved from Wageningen Economic Research (2004–2011) and the Netherlands Veterinary Medicines Authority (SDa) (2012–2020).

**Figure 2.**
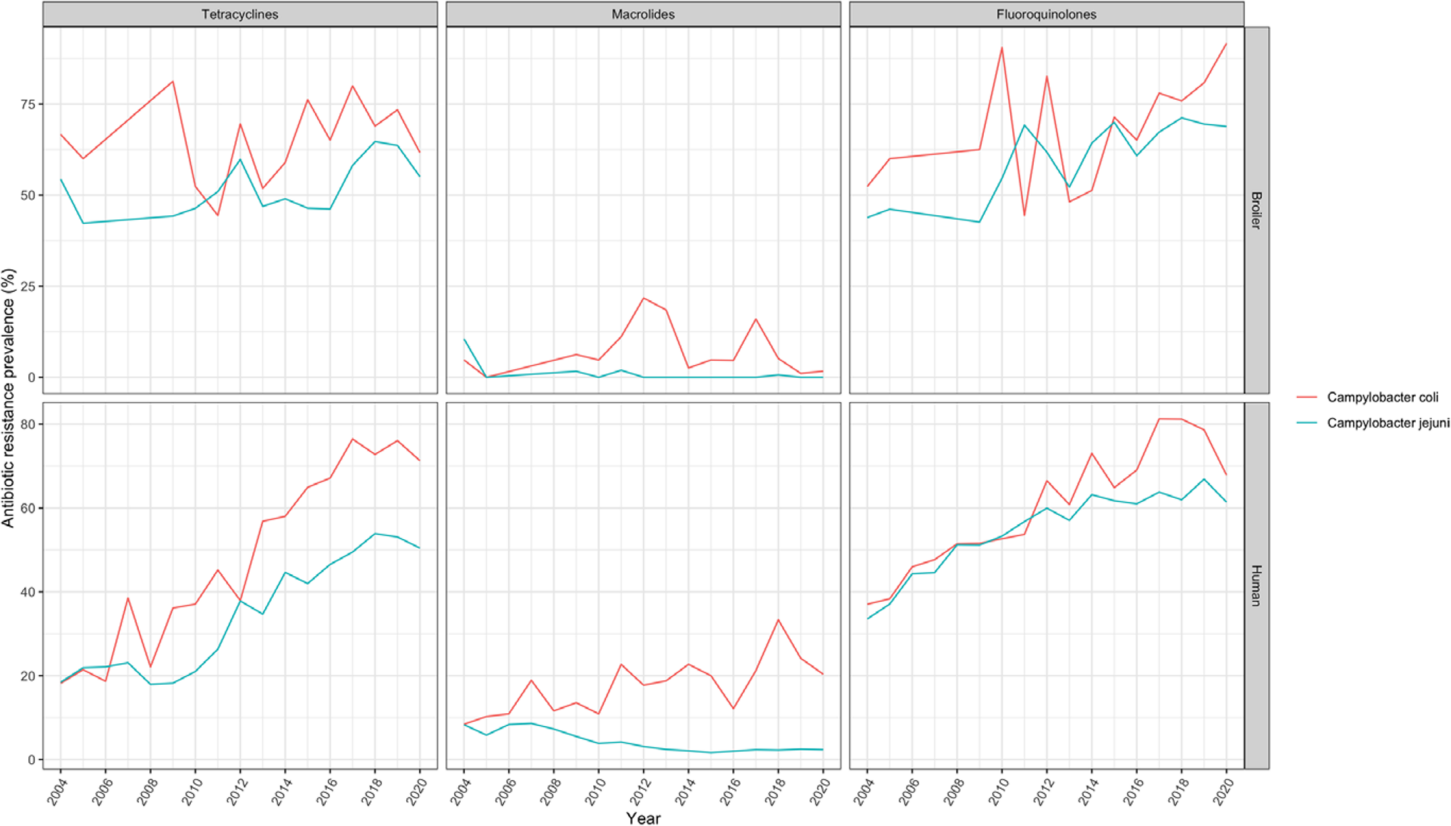
Annual prevalence of *Campylobacter jejuni*/*Campylobacter coli* isolates resistant to tetracyclines, macrolides, and fluoroquinolones from human campylobacteriosis cases and broilers at slaughter level in the Netherlands (2004–2020).

The trends in AMU (DDDA/Y) in different animal sectors are shown in Figure 1. In broilers, the total AMU increased by 161% from 2004 until 2009 (14.1 to 36.8) and then decreased remarkably by 75% until 2020 (9.3). In veal calves, the total AMU decreased by 55% from 2007 to 2020 (34.0 to 15.3). The total AMU in turkeys was less stable, but in general had a decreasing trend (−56%, 30.7 in 2010 to 13.6 in 2020). Overall, for individual antimicrobial classes, a relative decrease in AMU was notable over the study period in different animal sectors, especially for the usage of tetracyclines in broilers (−82%, from 5.5 in 2004 to 1.0 in 2020), veal calves (−57%, from 18.3 in 2007 to 7.8 in 2020), and turkeys (−37%, from 11.2 in 2013 to 7.1 in 2020) (Figure 1). The usage of macrolides and fluoroquinolones was stable at a low level in all livestock sectors. The usage of macrolides and fluoroquinolones in broilers and fluoroquinolones in veal calves was less than <0.5 DDDA/Y in most of the years and was therefore excluded from the analyses for estimating the association between AMU in livestock and AMR in humans. Due to limited annual AMR data points in turkeys, veal calves, dairy cattle, and other cattle, only the changes in broilers (2004, 2005, and 2009–2020) were explored and included in the analysis. In broilers, the prevalence of resistance against tetracyclines and fluoroquinolones in *C. jejuni*/*coli* isolates was stable at a high level, ranging from 42% to 81% and 43% to 92%, respectively. The resistance prevalence to macrolides was relatively low, ranging from 0% to 22% (Figure 2).

The percentages of resistance against tetracyclines, macrolides, and fluoroquinolones in *Campylobacter coli* and *Campylobacter jejuni* isolates from human cases showed increasing trends, except for resistance against macrolides in *C. jejuni* (Figure 2). The relative changes in prevalence of resistance in *C. coli* and *C. jejuni* isolates were + 75% and + 66% to tetracyclines, respectively, +72% and − 17% to macrolides, and + 45% and + 46% to fluoroquinolones.

Associations between AMU in livestock and AMR among isolates from human cases.

**Figure 3.**
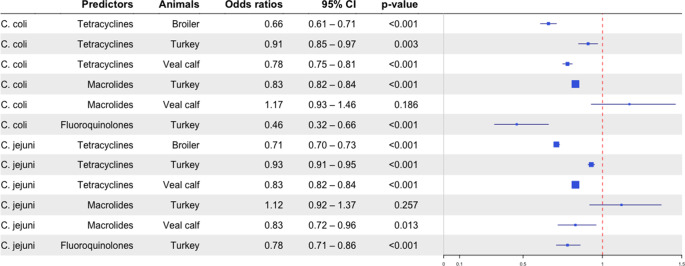
Associations between AMU in livestock and AMR in *Campylobacter jejuni*/*Campylobacter coli* isolates from human cases in the Netherlands (2004–2020). * The size of the blue boxes is based on precision.

The adjusted odds ratios with 95% CI for the associations between AMU in the specific livestock sector and AMR among human *C. jejuni*/*coli* infections are shown in Figure 3. Overall, statistically significant and inverse associations between AMU in livestock and AMR among human isolates were found in almost all antimicrobial classes, indicating that despite the decline in AMU in livestock, resistance in humans was still increasing. We found an indication for positive associations with macrolide-resistant *C. coli* from humans with the usage of macrolides in veal calves and turkeys, but the estimates were not statistically significant. Results from the sensitivity analysis with a one-year lag effect were similar to those of the main analysis and are therefore not presented.

Associations between AMU in broilers and AMR in isolates from broilers.

The association for AMU in broilers and AMR in *Campylobacter* isolates from broilers could only be investigated for tetracyclines because the usage of the other antimicrobial classes was below 0.5 DDDA in at least one of the years. The adjusted odds ratios for the associations between tetracycline usage in broilers and tetracycline resistance in isolates from broilers were 0.88 (95% CI: 0.74–1.05) for *C. coli* and 0.95 (95% CI: 0.87–1.04) for *C. jejuni.*

Correlations between AMR in broilers and AMR in isolates from humans.

The associations between yearly prevalence of AMR among *C. jejuni*/*coli* isolates in broilers and humans generally showed moderate to strong positive correlations (Table 1). The prevalence of resistance against tetracyclines and fluoroquinolones in broilers and resistance of corresponding antimicrobial classes in human *C. jejuni* isolates were significantly positively correlated.

**Table 1.** Correlations between AMR in *Campylobacter jejuni/Campylobacter coli* isolates from broiler and AMR in human *C. jejuni/coli* isolates from human campylobacteriosis cases in the Netherlands (2004–2020)

	AMR prevalence (%) in humans			
	Antimicrobial classes	*Campylobacter* species	ρ	*p*-value
AMR prevalence (%) in broilers	Tetracyclines	*C. jejuni*	0.7	**0.01**
		*C. coli*	0.3	0.30
	Macrolides	*C. jejuni*	0.3	0.37
		*C. coli*	−0.2	0.34
	Fluoroquinolones	*C. jejuni*	0.7	**0.003**
		*C. coli*	0.4	0.22

## Discussion

In this study, the associations between AMU in livestock (poultry and cattle) and AMR in *C. jejuni/ coli* from human infections were explored. Overall, the usage of tetracyclines, macrolides, and fluoroquinolones in those animals was found to be inversely associated with AMR in human *C. jejuni/coli* isolates.

Reducing veterinary AMU is expected to contribute to decreasing AMR among human infections in the long term, especially for infections caused by zoonotic pathogens, and this is noticeable in indicator bacteria such as *E. coli* [19]. However, evidence for zoonotic pathogens is scarce, and the few published studies have shown contrasting results in terms of effect size and direction of the relationship [20-23]. A meta-analysis including 13 studies that assessed the impact of AMU reduction in food animals on the prevalence of antibiotic-resistant bacteria (i.e., *Campylobacter* spp., *Enterococcus* spp., *Escherichia coli*, *Staphylococcus* spp.) in humans concluded that the pooled prevalence of AMR in human isolates was 24% lower in the intervention group, where the use of antibiotics in animals was reduced, compared with the control group [20]. Dutil et al. showed that the temporary suspension (in 2005) and reinstitution (in 2007) of ceftiofur usage in broiler chicken hatcheries in Québec, Canada, was associated with respective decreases and increases in ceftiofur-resistant *Salmonella* Heidelberg in samples from both chicken meat and humans [24]. A modelling study showed that curtailing the volume of antibiotics consumed by food animals, as a stand-alone measure, has little impact on the level of resistance in human infections [21]. A major drawback of these studies is that antimicrobial usage is usually unknown or poorly quantified and does not control for potential confounders (e.g., resistance to other antimicrobials (co-selection)).

In this study, longitudinal data on AMU and AMR covering 2004 – 2020 retrieved from national surveillance systems were used. We found inverse associations between AMU in livestock in the Netherlands and AMR in Dutch human *C. jejuni* /*coli* isolates (Figure 3). These outcomes are different from the findings of the JIACRA report, which investigated the impact of AMU in both human and animal sectors on the occurrence of AMR in these sectors in 2016 – 2018 by using data from the EU-wide surveillance programmes [19]. They reported a significant positive association between tetracycline and fluroquinolone usage in food-producing animals (expressed in mg per kg of estimated biomass/year) and resistance of *C. jejuni* from humans, although the associations for tetracycline were weak (odds ratios ranged from 1.01–1.02). No statistically significant associations between consumption of macrolides in food-producing animals and macrolide resistance of *C. jejuni* from humans were found [19]. The complexity of the antibiotic resistance problem, e.g. different bacteria-drug-animal combinations, co-selection of resistance due to the exchange of mobile genetic elements, surveillance data in different settings, and different analytical methods and outcomes often make the results difficult to compare.

Several aspects should be recognized when interpreting the inverse correlation found in our study. Firstly, AMR data collected from broilers in this study all originated from nationally produced animals. However, part of the human *Campylobacter* infections may result from the consumption of imported fresh meat, and AMR among the causative isolates may be the result of selection pressure imposed elsewhere. Since freezing is an effective way of reducing the concentration of *Campylobacter* in meat, imported fresh meat is the main concern [25]. The Netherlands imports poultry (both live animals and meat products) primarily from Germany, Belgium, the United Kingdom, Denmark, and France. The average occurrence of *Campylobacter* in the chilled broiler carcasses sampled in the EU and the Netherlands were 38.3% and 26.1% in 2022, respectively [26]. However, quantitative data of the trade volumes concerned is not available. Consequently, the exact contribution of imported fresh meat from neighbouring EU countries to AMR in *Campylobacter* isolates within the Netherlands is, therefore, unclear. Secondly, a national surveillance study among human campylobacteriosis cases in the Netherlands demonstrated that the prevalence of resistance to fluoroquinolones was higher in travel-associated infections (54%) compared to infections acquired domestically [27]. Similar results were also reported in Norway and Finland [28, 29]. Because the travel history of Dutch campylobacteriosis cases is unknown for the majority of the cases, part of the AMR among human isolates may be the result of selection pressure that occurred elsewhere, which might have influenced our results. Thirdly, even though human *Campylobacter* infections are mainly food-borne (especially from poultry and cattle) [30], there is evidence for other pathways, including contact with colonized animals (e.g., pets) and contaminated environments, as well as, rarely, people in conditions of poor hygiene [31-33]. It is challenging to take all the different routes and animal species into account. For example, the association of antibiotic usage in dairy cattle and other cattle to AMR in human isolates could not be assessed because of the low usage in some of the years (DDDA/Y < 0.5). Moreover, it was not possible to account for seasonality, urbanization degree, as well as regional differences within the country [27]. Besides the above-mentioned aspects, it remains unclear what mechanisms caused the inverse associations between AMU in livestock and AMR in Dutch human *C. jejuni/coli* isolates.

AMU in broilers has decreased dramatically since 2009 in the Netherlands. However, in our study, AMR in *Campylobacter* from poultry did not show any decreasing trend (Figures 1 and 2). Similar results were found in the JIACRA report, where no significant association was observed between tetracycline usage in poultry and resistance of *C. jejuni* in poultry [19]. However, positive associations were found for macrolides and fluoroquinolone usage [19]. Our results might be explained partly by the resistance mechanisms of *Campylobacter* [34]. For instance, *Campylobacter* isolates harbouring tetracycline resistance, conferred by *tet*(O) [35], appear to be widely distributed across various animal species and the environment [36]. Because *Campylobacter* can probably acquire *tet*(O) by horizontal gene transfer (HGT) from either *Streptomyces*, *Streptococcus*, or *Enterococcus* spp., it may not be directly related to AMU [37]. Furthermore, resistance to fluoroquinolones primarily arises from single point mutations in the quinolone resistance-determining region (QRDR) of DNA gyrase A (GyrA) [38]. Comparing to stepwise accumulation of several point mutations in other enteric organisms (e.g., *Salmonella* and *E. coli*), the resistance mechanism of *Campylobacter* leads to rapid development of fluoroquinolone-resistant mutants during antibiotic treatment [39, 40]. In contrast, the development of macrolide-resistant mutants requires a multistep process and prolonged exposure to the macrolide antibiotics, which is one of the mechanisms contributing to the relatively low prevalence of macrolide resistance in *Campylobacter* [41]. Despite a substantial reduction in AMU, particularly in tetracyclines, and stable low-level usage of macrolides and fluoroquinolones in broilers, the prevalence of resistance to these antimicrobials in *C. jejuni* and *C. coli* remains high. This persistence may be due to natural selection on resistance or related to previous antimicrobial usage in broilers, preserving resistant *Campylobacter* isolates in the farm environment even during periods of low AMU. A recently published study also showed that no associations were noted between the resistance and use of the same antimicrobial in Canadian turkey flocks, but the use of certain antimicrobial classes may have played a role in the maintenance of resistance in *Campylobacter* [42]. Fluoroquinolone-resistant *Campylobacter* may not have a fitness disadvantage and even may have a fitness advantage compared to susceptible strains [43]. Therefore, while reducing AMU in broilers may exert some influence, its impact on AMR in isolates from these animals may be moderate. It is important to note that this is an ecological study using aggregated data at the country level to generate hypotheses of associations between AMU in livestock and AMR in humans. No matter how strong the associations are, causation cannot be confirmed by this study type. Results should, therefore, be interpreted with caution as they might be biased by the absence of variation over aggregated data (i.e., ecological fallacy).

Our results showed that there were moderate to strong positive correlations between the prevalence of tetracyclines and fluoroquinolone resistant *C. jejuni* isolates from broilers and resistance to the same antimicrobials in human *C. jejuni* isolates (Table 1). This indicates that AMR in human isolates increases with increased AMR in animal isolates, and this agrees with the findings from previous studies [19, 20, 23]. The main identified source of human *Campylobacter* infections in the Netherlands is contaminated broiler chicken meat. Therefore, reducing contamination of fresh chicken meat entering the kitchen and enhancing hygienic measures in the kitchen might be one of the most efficient intervention measures for reducing the disease burden of *Campylobacter.*

To conclude, we observed that the substantial reduction in livestock AMU achieved in the Netherlands in recent years does not seem to be temporally associated with reduced AMR among isolates from human campylobacteriosis cases. Our results also showed that resistance against tetracyclines and fluoroquinolones in broilers and resistance of corresponding antimicrobial classes in human *C. jejuni* isolates were significantly positively correlated. These results suggest that reducing AMU in Dutch livestock alone might not suffice in significantly reducing AMR among *Campylobacter* isolates, at least not in the short term, and preventing (zoonotic) transmission of *Campylobacter* in general, not AMR per se, may be more effective as a strategy. Due to the ecological study design, we were unable to make direct links between the selection of AMR in *Campylobacter* isolates from human cases and the impact of various exposure factors. Further analyses need to consider other factors at play, e.g. consumption of imported fresh meat and travel history of patients, AMU in humans, infections via other pathways, such as contact with colonized animals (e.g., companion animals) and contaminated environments, to better understand the observed effects.

## Data Availability

The data that support the findings of this study are available from the corresponding author, upon reasonable request.

## Members of the ISIS-AR study group

J.W.T. Cohen Stuart, Noordwest Ziekenhuisgroep, Department of Medical Microbiology, Alkmaar.

D.C. Melles, Meander Medical Center, Department of Medical Microbiology, Amersfoort.

K. van Dijk, Amsterdam UMC, University of Amsterdam, Department of Medical Microbiology and Infection Prevention, Amsterdam Infection and Immunity Institute, Amsterdam.

A. Alzubaidy, Atalmedial, Department of Medical Microbiology, Amsterdam.

M. Scholing, OLVG Lab BV, Department of Medical Microbiology, Amsterdam.

S.D. Kuil, Public Health Service, Public Health Laboratory, Amsterdam.

G.J. Blaauw, Gelre Hospitals, Department of Medical Microbiology and Infection prevention, Apeldoorn.

ISIS-AR Projectteam: W. Altorf - van der Kuil, S.M. Bierman, S.C. de Greeff, S.R. Groenendijk, R. Hertroys, J.C.M Monen, D.W. Notermans, J. Polman, W.J. van den Reek, C. Schneeberger-van der Linden, A.F. Schoffelen, F. Velthuis, C.C.H. Wielders, B.J. de Wit, R.E. Zoetigheid, Centre for Infectious Disease Control (CIb), National Institute for Public Health and the Environment (RIVM), Bilthoven, The Netherlands.

W. van den Bijllaardt, Microvida Amphia, Laboratory for Microbiology and Infection Control, Breda.

E.M. Kraan, IJsselland hospital, Department of Medical Microbiology, Capelle a/d IJssel.

M.B. Haeseker, Reinier de Graaf Group, Department of Medical Microbiology, Delft.

J.Macaria da Silva, Deventer Hospital, Department of Medical Microbiology, Deventer.

E. de Jong, Slingeland Hospital, Department of Medical Microbiology, Doetinchem.

B. Maraha, Albert Schweitzer Hospital, Department of Medical Microbiology, Dordrecht.

A.J. van Griethuysen, Gelderse Vallei Hospital, Department of Medical Microbiology, Ede.

B.B. Wintermans, Admiraal De Ruyter Hospital, Department of Medical Microbiology, Goes.

M.J.C.A. van Trijp, Groene Hart Hospital, Department of Medical Microbiology and Infection Prevention, Gouda.

A.E. Muller, Haaglanden MC, Department of Medical Microbiology, ’s-Gravenhage.

M. Wong, Haga Hospital, Department of Medical Microbiology, ’s-Gravenhage.

A. Ott, Certe, Medical Microbiology Groningen|Drenthe, Groningen.

E. Bathoorn, University of Groningen, University Medical Center, Department of Medical Microbiology, Groningen.

M. Lokate, University of Groningen, University Medical Center, Department of Medical Microbiology, Groningen.

J. Sinnige, Regional Public Health Laboratory Haarlem, Haarlem.

D.C. Melles, St Jansdal Hospital, Department of Medical Microbiology, Harderwijk.

N. Plantinga, Laboratory of Medical Microbiology and Public Health, Hengelo.

N.H. Renders, Jeroen Bosch Hospital, Department of Medical Microbiology and Infection Control, ’s-Hertogenbosch.

J.W. Dorigo-Zetsma, CBSL, Tergooi MC, Department of Medical Microbiology, Hilversum.

L.J. Bakker, CBSL, Tergooi MC, Department of Medical Microbiology, Hilversum.

W. Ang, Comicro, Department of Medical Microbiology, Hoorn.

K. Waar, Certe, Medical Microbiology Friesland|NOP, Leeuwarden.

M.T. van der Beek, Leiden University Medical Center, Department of Medical Microbiology, Leiden.

M.A. Leversteijn-van Hall, Eurofins Clinical Diagnostics, Department of Medical Microbiology, Leiden-Leiderdorp.

S.P. van Mens, Maastricht University Medical Centre, Department of Medical Microbiology, Infectious Diseases and Infection Prevention, Maastricht.

E. Schaftenaar, St Antonius Hospital, Department of Medical Microbiology and Immunology, Nieuwegein.

M.H. Nabuurs-Franssen, Canisius Wilhelmina Hospital, Department of Medical Microbiology and Infectious Diseases, Nijmegen.

I. Maat, Radboud University Medical Center, Department of Medical Microbiology, Nijmegen.

P.D.J. Sturm, Laurentius Hospital, Roermond.

B.M.W. Diederen, Bravis Hospital, Department of Medical Microbiology, Roosendaal.

L.G.M. Bode, Erasmus University Medical Center, Department of Medical Microbiology and Infectious Diseases, Rotterdam.

D.S.Y. Ong, Franciscus Gasthuis and Vlietland, Department of Medical Microbiology and Infection Control, Rotterdam.

M. van Rijn, Ikazia Hospital, Department of Medical Microbiology, Rotterdam.

S. Dinant, Maasstad Hospital, Department of Medical Microbiology, Rotterdam.

M. den Reijer, Star-SHL, Rotterdam.

D.W. van Dam, Zuyderland Medical Centre, Department of Medical Microbiology and Infection Control, Sittard-Geleen.

E.I.G.B. de Brauwer, Zuyderland Medical Centre, Department of Medical Microbiology and Infection Control, Sittard-Geleen.

R.G. Bentvelsen, Microvida ZorgSaam, Department of Medical Microbiology, Terneuzen.

A.G.M. Buiting, St. Elisabeth Hospital, Department of Medical Microbiology, Tilburg.

A.L.M. Vlek, Diakonessenhuis, Department of Medical Microbiology and Immunology, Utrecht.

M. de Graaf, Saltro Diagnostic Centre, Department of Medical Microbiology, Utrecht.

A. Troelstra, University Medical Center Utrecht, Department of Medical Microbiology, Utrecht.

K.B. Gast, Eurofins-PAMM, Department of Medical Microbiology, Veldhoven.

M.P.A. van Meer, Rijnstate Hospital, Laboratory for Medical Microbiology and Immunology, Velp.

J. de Vries, VieCuri Medical Center, Department of Medical Microbiology, Venlo.

J.D. Machiels, Isala Hospital, Laboratory of Medical Microbiology and Infectious Diseases, Zwolle.
