# Reducing antimicrobial use in livestock alone may be not sufficient to reduce antimicrobial resistance among human Campylobacter infections: an ecological study in the Netherlands – CORRIGENDUM

**DOI:** 10.1017/S0950268826101290

**Published:** 2026-04-06

**Authors:** Huifang Deng, Linda E. Chanamé Pinedo, Anouk P. Meijs, Pim Sanders, Kees T. Veldman, Michael S. M. Brouwer, Wieke Altorf-van der Kuil, Bart Wullings, ISIS-AR Study Group, Maaike J. C. van den Beld, Sabine C. de Greeff, Cindy M. Dierikx, Engeline van Duijkeren, Eelco Franz, Lapo Mughini-Gras, Roan Pijnacker

When this article was published in Epidemiology & Infection it contained an error in the name of the author Wieke Altorf-van der Kuil. This has since been updated. The authors apologise for this error.
